# English Language Teacher Agency in Response to Curriculum Reform in China: An Ecological Approach

**DOI:** 10.3389/fpsyg.2022.935038

**Published:** 2022-07-18

**Authors:** Lian Wang

**Affiliations:** School of Foreign Languages, Guizhou University of Finance and Economics, Guiyang, China

**Keywords:** ecological approach, teachers' agency, curriculum reform, educational changes, teaching practices

## Abstract

This study draws on the ecological perspective of teacher agency to examine the manifestation of English teachers' agency toward the ongoing curriculum reform in China and the factors that impact it. This study surveyed 353 high school English teachers and then collected data from three case study participants through in-depth interviews. The findings showed that the majority of teachers surveyed exhibited positive attitudes and beliefs about implementing the reform and inclinations to change, but the teachers also showed a constrained state of agency in practice. Teacher agency developed as the teachers exerted sustained pedagogical change and reflection on reform-based practices. Through the findings, prior experiences and reform-oriented beliefs were found to mediate teachers' agency, and reform-related experiences were more influential than future goals in shaping agency. The factors of perceived school culture that involved teachers' interaction with students, colleague cooperation, and administrative support also medicated teachers' agency in practice. Implications are proposed for policymakers and school leaders to help teachers coordinate inconsistencies between high-stakes examination preparation and holistic education and make positive sense of professional development in the context of educational changes.

## Introduction

Teacher agency is imperative for the process of implementing curriculum reforms and educational policies (Lasky, 2005; Pyhältö et al., 2012; Hamid and Nguyen, 2016; Tao and Gao, 2017). Current empirical studies show that teacher agency is a temporal and situated achievement and that teachers exercise their agency to respond to educational change in different manifestations, such as compliance, resistance, and negotiation (Robinson, 2012; Priestley et al., 2015; Yang and Clarke, 2018; Le et al., 2020). These studies constitute an area of inquiry of growing importance, which is known as teacher agency research in policy implementation, and they reveal that personal factors (e.g., teachers' beliefs) and contextual factors (e.g., teaching contexts) contribute significantly to teachers' change and growth during curriculum reform. Nevertheless, notably, teachers' agency development during educational changes is the outcome of the interplay within their professional life experiences and the expectations for future and contextual conditions.

A number of studies reveal that teachers are more likely to resist imposed policy mandates and embrace conventional teaching techniques to avoid taking risks in educational reform if the mandates are incongruous with their personal factors, such as their beliefs, intentions, and prior experiences (Biesta et al., 2015; Bonner et al., 2019). Le et al. (2020), for instance, emphasize that English teachers are more likely to exercise their agency to struggle and resist educational language policy when there is a conflict between policy mandates and their beliefs, prior knowledge, and expectations. Teachers' agentic actions toward educational change are always mediated by the sociocultural contexts in which they are situated. Sociocultural contexts are reflected not only in classroom teaching, school conditions, and local communities but also in educational policy mandates, the promotion of ideological discourses, and changes in assessment practice that can either enable or hinder teachers' agency enactment (Lasky, 2005; Priestley et al., 2015; Poulton, 2020; Tao and Gao, 2021). For example, Liyanage et al. (2015) identified the struggles and dilemmas experienced by English language teachers in their attempts to exercise agency amid the instructional demands of the exam-oriented community in Inner Mongolia in China. Since teacher agency's interaction with other personal and contextual factors does not operate alone, a focus on teacher agency during curriculum reform should not distract researchers from profoundly examining the integration of their historical experiences, present conditions, and future aspirations that constitute an ecological perspective to explain how teachers make agentic choices and actions during educational changes. In China, the Ministry of Education (MOE) enacted the 2017 Edition of the General High School English Curriculum Standards (课程标准, also known as *xin kebiao)* in 2018, which was initiated to cultivate learners' four key competencies, namely language competence, cultural awareness, thinking capacity, and learning capacity, as the core competencies (核心素养, hexin suyang) in basic English as a Foreign Language (EFL) education. Therefore, this study goes inside the classroom teaching in mainland China and investigates how Chinese English teachers' agency in response to the prescribed national curriculum may be interpreted, and it takes an ecological approach with reference to the factors that enable or constrain teacher agency during educational reforms.

English is the foreign language studied by more than 99% of students who participate in formal education in China (Wang, 2007). Although English language teaching (ELT) and assessment in China has experienced a considerable transition in recent decades, ELT has continued to rely on a tradition of centrally administered exams to determine achievement and opportunity, and constraints continue to operate as major structural elements of the English teaching environment in China (Liyanage et al., 2015). In combatting the chronic malady of “only scores” and “only further education,” the advert of *xin kebiao* is considered a driving force for a new round of English curriculum reform in China, and the new syllabus gives teachers guiding principles and pedagogical approaches, such as the activity teaching method, to teaching in action, which challenges teachers to take more responsibilities in innovating professional skills and nurturing quality education. Meanwhile, notably, the implementation of educational policies in China follows a traditional centralized and top-down manner in which prescriptive policies are initiated and imposed at the macrolevel (i.e., the MOE). Although a few studies have explored Chinese university teachers' agency and their professional development in educational reforms (Tao and Gao, 2017; Yang and Clarke, 2018; Tao et al., 2020), more research is needed to examine how English teachers from different high schools teach in accordance with the core competencies (hexin suyang) in response to the prescribed national curriculum and the extent to which they might exert positive agentic changes in the context of China.

## Teacher Agency in Curriculum Reform

Research has noted that there is often a tension in educational policies about the amount of control that teachers are allowed to exert over the curriculum and what and how they teach (Hamid and Nguyen, 2016; Poulton, 2020). That is, implementing innovation with an educational reform is not a matter of straightforwardly executing policies; rather, it involves a process of sense-making through which teachers make meaning from their work environment (Vähäsantanen, 2015). Regarding English language teachers, a few studies have explored teacher agency during educational changes (Hamid and Nguyen, 2016; Tao and Gao, 2017; Le et al., 2020). Among them, Nguyen and Bui (2016) emphasize that teachers do not passively follow a set of norms mandated by policymakers but act as agents in shaping and reshaping a language policy through their pedagogical practices. It has been noted that a positive association was found between teachers' agency and their engagement with research and reflection on teaching in the context of the Chinese national college English reform (Yang and Clarke, 2018). The studies of Li et al. (2020) showed that English primary teachers in Vietnam attempted to adapt the new language policy mandates according to their interpretation, preferences, choices, and current teaching conditions. One noticeable contribution of these empirical studies is that English teachers can act as agents for curriculum reform, in which the dynamic development of teachers' professional practices can be partly explored through their different agency commitments. As agency in educational changes is practiced as a mediational tool that is crucial for teachers to resolve structural conflicts among personal beliefs, language policies, and hierarchical power relationships (Liyanage et al., 2015; Yang and Clarke, 2018), the research on English language teacher agency is, despite the growing interest in recent years, still modest in the current language policy literature.

## Defining Teacher Agency

The notion of agency has long been researched from quite different theoretical perspectives. There is a lack of consensus on the conceptualization of agency. Specifically, agency has been viewed as individuals' intentional acts to make things happen and participate in their development, adaptation, and self-renewal with changing times (Bandura, 2001). This concept emphasizes the psychological mechanism of the self-system in one's agency formation, while some scholars have argued that agency is a socioculturally medicated capacities to act (Ahearn, 2001; Lasky, 2005; Kayi-Aydar, 2019). Goller and Harteis (2017) derived a definition of agency on both a psychological and practical basis suggesting that human agency is the capacity and tendency to make intentional choices, initiate actions based on these choices, and exercise control over the self and the environment. More recent attempts have been made to define agency in an ecological term as “a matter of personal capacity to act, combined with the contingencies of the environment within which such action occurs” (Priestley et al., 2012). The ecological perspective of agency argues the notions that agency is the capacity and tendency to make intentional choices, initiate actions based on these choices, and exercise control over the self and the environment (Eteläpelto et al., 2013). These notions suggest that even if actors have some type of capacity, whether they can achieve agency depends on the interaction of the capacities and the ecological conditions. Actors always act through an environment rather than simply in an environment (Biesta and Tedder, 2007; Priestley et al., 2015).

**Figure 1:**
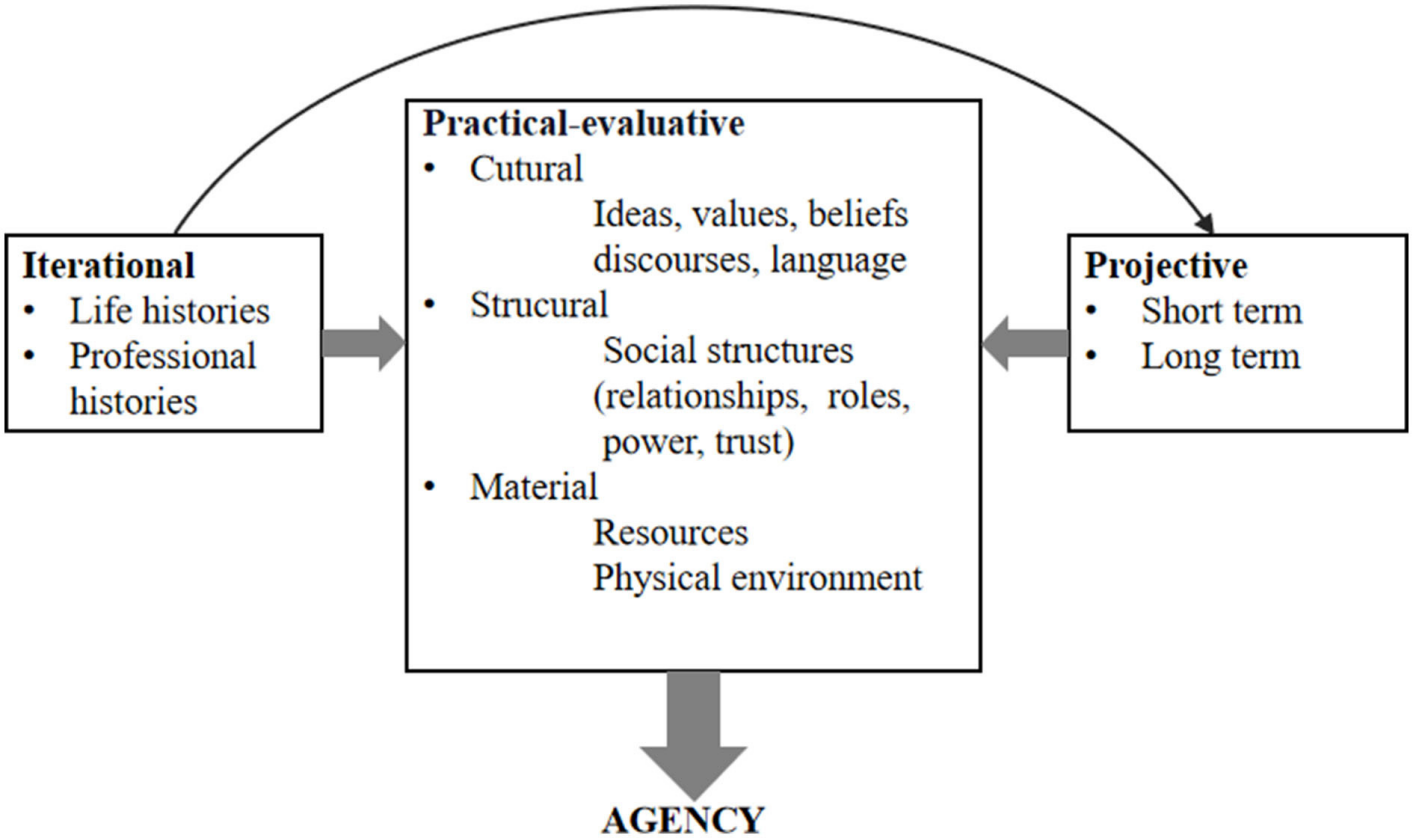
Model of the formation of agency. Redrawn from Priestley et al. (2015), with permission from Bloomsbury Academic through PLS Clear.

Agency has also been researched in the field of teacher education. For teachers, it is largely about repertoires for maneuvering or the possibilities for different forms of action available to them at particular points in time (Priestley et al., 2012). Therefore, Priestley et al. (2015) proposed an ecological perspective to acknowledge that teacher agency, as a temporal situated achievement, contains the three dimensions: iterational, practical-evaluative, and projective (see Figure 1). Language teachers, as active agents in social and educational contexts, directly and indirectly, experience their professional qualities as being naturally shaped by the environment in which they live (Chu et al., 2021). In this regard, teacher agency in response to curriculum reform may be afforded or constrained as the outcome of the interplay among an individual's past experiences, present conditions, and future goals.

The ecological perspective has been considered an effective approach for understanding the phenomenon of one's agency not only in context but also in one's life history (Tao and Gao, 2021). This model offers a more explicit ecological approach to teacher agency that constitutes both a methodological and a theoretical framework for empirical inquiry that relates to the approaches by which teachers achieve agency in practice. Agency is not simply concerned with the ways in which we engage with our contexts-for-action but rather with the capacity to shape our responsiveness to the situations that we encounter in our lives (Biesta and Tedder, 2007). The ecological approach provides useful insight into how teacher agency can be located through a consideration of their histories and beliefs, their abilities to visualize the alterative future, and their interplay with the sociostructural and material conditions in which individuals act (Priestley et al., 2015). Since the ecological perspective allows a historical perspective, Tao and Gao (2017) examined language teachers' professional trajectories in which their agency was situated by life-history exploration. Framed within an ecological conceptualization of teachers' agency, Poulton (2020) explored Australian primary teachers' reported experiences of agency and identified potential enablers and constraints to teachers' agency in curriculum planning and teaching; they found that strong beliefs, teacher knowledge, and skill and aspirations for school-based assessment helped teachers report greater experiences of agency. Liu's et al. (2022) study highlighted that English teachers, as active agents situated in the educational context, could positively negotiate with their surroundings, and exert their agency to cope with professional and personal pressures. To conclude, the exploration of teacher agency has gained much empirical support and demonstrated diverse theme, while EFL teacher agency in response to curriculum reform has not been thoroughly examined from both psychological and ecological perspectives. In this context, mainly drawn on the ecological model of teacher agency proposed by Priestley et al. (2015), this study aims to adopt a mixed-method design to enrich the understanding of EFL teacher agency in response to curriculum reform in China by addressing the following questions:

(1) In what way do teachers exercise agency in response to top-down curriculum reform?

(2) What are the factors that enable and constrain teacher agency during curriculum reform?

(3) How do these factors interact with teacher agency to facilitate the sustained implementation of the reform?

## Methods

The study employed a two-phased, sequential mixed methods explanatory design framework (Cresswell and Plano Clark, 2007). Precisely, the method used for the initial phase of the study was a survey of Chinese high school English teachers' agentic performance during the reform. For this purpose, a questionnaire has been constructed based on Goller and Harteis (2017)'s perceptions of agency constitutions. The questionnaire formulated three dimensions of teacher agency: intention level, action level, and regulation level. Intention level refers to teachers' action plans and strategies for participating in the reform. Action level refers to teachers' self-initiated and goal-directed behaviors that aim to take control over themselves and the environment. Regulation level refers to teachers' self-motivation and self-reflectiveness in light of educational change. The questionnaire consists of questions, such as “Do you agree that the core competencies should be integrated into actual teaching practice?”, “Do you often consciously nurture students' thinking capacity in class activities?”, “Do you often exchange teaching experience with colleagues about the core competencies?”, “Do you often reflect on your teaching process and adjust actions in line with *xin kebiao*?”, etc. To investigate the factors that had an impact on teachers' agency, this study also developed an instrument to examine the influential factors on teacher agency from the three established dimensions of participants' demographic characteristics, their reform-oriented beliefs, and their perceived school culture. The questionnaire uses a Likert scale from 1 (totally disagree or have never done) to 5 (absolutely agree or have always done).

Overall, the established questionnaire has four parts that consist of participants' demographic variables (teaching age, gender, education level, professional title, and school type), items concerning teachers' agency (*n* = 25), items concerning teachers' reform-oriented beliefs (*n* = 7), and items concerning teachers' perceived school culture (*n* = 13). The survey items and instructions were revised and validated in a pilot test before delivering the final questionnaire by using SPSS 21.0 and AMOS 7.0 in the aspects of item analysis, validity, and reliability analysis. Item analysis was first performed to detect the discriminant validity of each item. After a series of adjustments, five items were deleted from the scale of teacher agency, and three items were deleted from the scale of teacher-perceived school culture. Since the factor structure drew on previous conceptualizations of agency (Goller and Harteis, 2017), a confirmatory factor analysis (CFA) was adopted to examine the goodness of fit of the items from the teachers' agency scale. The CFA showed that the load values of 5 items included in the intension variable ranged from 0.65 to 0.76 on this factor, the load values of 7 items in the action variable ranged from 0.61 to 0.73 on this factor, and the load values of 8 items in the regulation variable ranged from 0.61 to 0.80 on this factor. The reported degree of the fitting indices of the three variables was acceptable as^2^/df = 2.488, CFI = 0.927, PCFI = 0.815, RMSEA = 0.065. These results indicated that 20 items from the revised scale of teacher agency were well-supported. Regarding teacher-perceived school culture, both an exploratory factor analysis (EFA) and CFA were used to examine the factor structure, and a two-factor structure composed of interpersonal conditions and the school system was obtained to identify it. The KMO measure and Bartlett's test of sphericity were conducted to ensure that the data were eligible for factor analysis. The KMO index was 0.856, and Bartlett's test of sphericity (Approx.Chi-Square = 572.725, df = 45) was significant at a level of 0.000. The varimax method showed a high correlation between the items and the common factors, with all factor loadings >0.5 and commonality >0.4. Moreover, a CFA for each factor was conducted, and one item was removed from the teacher-perceived school culture dimension with a loading lower than 0.40. The CFA results indicated that 9 items of the adjusted scale of teacher-perceived school culture were much better than the initial scale (^2^/df = 1.847, CFI = 0.960, PCFI = 0.818, RMSEA = 0.049). After the CFAs, an internal consistency reliability analysis was used to assess each of the subscales. The alphas were 0.890 for teachers' agency, 0.889 for reform-oriented beliefs, and 0.892 for perceived school culture. The pilot results helped to revise the total items from 45 to 36 and established the final questionnaire for the participants (*n* = 387).

Based on convenience sampling strategies, the questionnaire was written in Chinese and distributed to teachers from different high schools in five provinces of China (Jiangsu, Hebei, Guizhou, Guangdong, and the Guangxi Zhuang Autonomous Region). A total of 353 questionnaires were collected, and the proportion of cleared samples was 91.2%, with 84 from Jiangsu, 77 from Hebei, 57 from Guizhou, 36 from Guangdong, and 99 from the Guangxi Zhuang Autonomous Region. In addition, the gender distribution of the convenience sample (male = 73, female = 280) was in accordance with the fact that male English teachers are largely outnumbered by female English teachers in China.

Within the second phase of the study, a multiple case study with three participants was adopted to explore how these potentially influential factors interact with teacher agency to facilitate the sustained implementation of this reform, which allowed an in-depth understanding of the participants' perspectives, beliefs, and action regarding educational changes. Three participants who were born in the 1970's, 1980's, and 1990's were all involved in the initial survey and voluntarily agreed to participate in the second phase of the study. They were from different schools, and their EFL teaching experience varied from 7 to 26 years, which demonstrated that their educational background, teaching proficiency, and professional development conditions were very different. Table 1 describes the demographic characteristics of the three participants.

**Table 1:** Profile of the case study participants.

**Characteristic**	**Participants**		
	**Zhang**	**Xu**	**Wang**
Gender	Female	Male	Female
Age	48	38	29
Teaching age	26 years	16 years	7 years
Grades	Grade 2	Grade 2	Grade 3
Qualifications	Master	Bachelor	Bachelor
Professional titles	Senior	Senior	Intermediate

## Data Analysis

To answer RQ1, the questionnaire data were analyzed quantitatively with SPSS 21.0 for descriptive analysis and variance analysis. To answer RQ2, a one-way analysis of variance (ANOVA) and regression were used to examine the correlations between teachers' agency and their background information, beliefs toward the reform, and perceived school culture. To answer RQ3, qualitative data were collected that involved the participants' reform-oriented beliefs, prior experiences, future goals, in-class teaching decisions, and actions related to curriculum reforms through semi-structured interviews. Interviews consisted of questions, such as “Have you ever taken a part in a national curriculum reform in the past, and can you describe its impacts on your teaching practice?”, “Can you describe the teaching objectives in your classes at present?”, etc. All these face-to-face interviews were conducted with each participant in their actual daily workplaces in an ongoing and cyclical process over 6 months. Drawn on the across-case analysis of qualitative study, categories and patterns were inductively generated (Miles and Huberman, 1994). Drawing on the model of the ecological approach to teacher agency, influential factors of the iterational, practical-evaluative and projective dimensions on their enactment of agency during this reform were identified in the initial reading and coding of the data. In particular, the three integrated elements of teachers' agency mentioned above guided the whole encoding work regarding the choices and actions of the pedagogical changes made by the participants in practice.

## Results

### EFL Teachers' Agency in Curriculum Reform

The descriptive statistics for the teachers' agency scores based on the scale showed that the mean value of teachers' agency to implement the reform is 4.063 in total. The results appear to suggest that most English teachers advocate for the mandates proposed in *xin kebiao*. Although this finding reveals the big picture of teachers' supportive perspectives of the reform, in discussing teacher agency, it is a question of not only how teachers position themselves in relation to reforms but also how they act in engaging with the reforms (Vähäsantanen, 2015). To have a close examination of teachers' agentic changes in accordance with policy-related demands, this part conducts a contrastive analysis of Wang's, Xu's and Zhang's agency.

#### Wang: Constrained Agency

In the interviews, Wang mentioned that her motivation to take on new pedagogical practice was not strong. She stated that compared with her colleagues in the English department, she was less capable of interpreting educational theories of the new syllabus into effective teaching practices. As she claimed, she did try to enact some of the innovative ideas and new teaching methods recommended in *xin kebiao*, but the interactions between the students and her turned out to be unsuccessful. Thus, she rested on traditional pedagogies by promoting word rehearsal, sentence translation, and grammatical drilling in class, which she believed to be helpful for the students in preparation for *GaoKao*, a high-stakes exam used to determine the selection to study at prestigious key universities, ordinary universities, colleges, or other higher education institutes (Fang and Warschauer, 2004). In Wang's case, she demonstrated a constrained state of agency for engagement in the reform. On the one hand, she struggled considerably with a sense of powerlessness for the frustrated adoption of new teaching methods in class; on the other hand, she made great efforts to fulfill her accountability for students' test-based performance to such an extent that her teaching practices were identical to her testing practice (Liyanage et al., 2015), which is contrary to the recommended teaching methodology in policy. In addition, Wang said she often “searched for professional assistance from experienced colleagues in deeply interpreting the connotation of some educational theories.” It may be inferred that Wang's minimal efforts to perform changes in practice could be explained by her resistance to taking risks at the cost of exam scores.

##### Extract 1

Wang: I designed some teaching activities guiding students to guess the meanings of words in contexts, but they couldn't answer my questions and were not interested in these activities, let alone fostering their thinking capacity. The educational ideas and goals recommended in *xin kebiao* are so good for students, but currently, the main goal in my classroom is to practice and memorize words and collocations. Thus, the meaning of texts can be understood.

#### Xu: Transformative Agency

Xu mentioned that given the competitive exam-based assessment system in the context of China, it was difficult to fully cultivate key competencies among students. Simultaneously, he felt obliged to improve his teaching practice and competence by changing beliefs, pedagogies, and practices in the classroom and maintaining a balance between test goals and holistic educational goals. Xu pointed out in interviews that the new syllabus focused on language skills in *listening, speaking, reading, writing*, and *watching*. For *watching*, he actively brought varying “Thinking maps” to innovate pedagogical practices to thus shift class practices from a grammar-translation approach to a more interactive approach. This teaching method is consistent with the ideas advocated in the reform. It seemed that Xu demonstrated a transformative state of agency in response to the reform. Faced with conflicts and dilemmas between public exam-oriented mindsets and holistic education required by the reform, Xu enacted an ongoing transformation of teaching expertise exempt from routine work to achieve innovations. These reform-based experiences that accompanied reflection constructed his practical knowledge and laid a solid foundation for his class practices, teaching contests, and pedagogical research. Xu talked about his impact on the students: “I always imparted new ideas reflected in examination to the students; they were good at problem-solving and critical thinking skills while aligning with academic standards.” Notably, a well-interactive relationship with students helped Xu make sense of his agency to enact pedagogies that he believed to be valuable for student learning.

##### Extract 2

Xu: I acknowledge that scores are always of the primary importance for students, most of their time and energy are put into the preparation for exams due to the present examination-success-oriented context, and very limited space of education has been left for students' all-around development. However, I always possess the capability to make decisions on my own, so I keep reflecting on how to infiltrate the core competencies recommended in *xin kebiao* into my class and concentrate more on developing students' cultural awareness and critical thinking capacity.

#### Zhang: Progressive Agency

As the head of the English department, the master teacher of beginning teachers, and the leader of the teaching workshop at school, Zhang has always kept learning on advanced curricular theories and embracing educational changes to enhance her professional expertise. She indicated that students' English learning and academic achievements could be greatly enhanced if innovative, flexible, and appropriate pedagogies were adopted in practice. She put considerable energy into learning and thinking critically about *xin kebiao* since the government enacted the policy. Specifically, she kept trying varied teaching approaches to explore effective practices of educational theories contained in *xin kebiao* with self-confidence. She spoke with evident enthusiasm about conducting reform-based research associated with pedagogical changes that innovated her practical teaching and achieved well in meeting her students' needs in creative ways, through which her conviction and responsibilities as curriculum developer strengthened greatly and motivated her to become one of the first adopters of the reform. To shift her role as a facilitator instead of being an authoritative instructor in class, Zhang cultivated students' capability of active learning by engaging them in self-assessment and peer assessment. Moreover, Zhang showed progressive agency with leadership in her school community: “I felt obliged to facilitate young unexperienced teachers in school to develop.” Zhang enacted her agency to negotiate her identity in her classroom with her students and within the school with colleagues; then, she took control of what was happening around her in the reform.

##### Extract 3

Zhang: I was once sent to participate in a national reform-based teaching competition on behalf of Guangxi Zhuang Autonomous Region. At the beginning, I felt stressed because the new syllabus only sets very general goals for English learning and teaching. It's my work to determine which teaching approaches, such as “task-based instruction,” “communicative language teaching,” “activity teaching method,” and “san yi san xiang method,” will be contextually appropriate for accomplishing my teaching objectives. Although preparing for the competition was laborious and painstaking, it was worth trying. I gained a lot during the process.

The manifestations of Wang's, Xu's, and Zhang's agency toward this reform at the individual scale can symbolize certain typical scenes and cases during educational changes in China. To summarize, the three teachers all showed positive perceptions of *xin kebiao* and its ideas, but they also exhibited different agentic changes in practice. In contrast with Wang's weak engagement with the reform, Xu and Zhang demonstrated critical shifts in response to the reform by constructing mutual support with students and sharing valuable resources with peer teachers.

### Demographic Characteristics and Reform-Oriented Beliefs

A series of one-way ANOVA tests were performed to establish whether there were significant differences in the respondents' demographic characteristics. The statistical analysis reported that no significant difference was found in divided age groups (*p* = 0.273 > 0.05) and layered educational groups (*p* = 0.261 > 0.05) on their agency values. However, the one-way ANOVA indicated that all groups of varied professional titles manifested significant differences (F = 4.41, *p* = 0.005). As seen in Table 2, the median of senior teachers' agency ranked the highest (m = 4.72), followed by associate senior teachers (m = 4.10), intermediate teachers (m = 4.09), and primary teachers (m = 3.99). This finding suggests that teachers with higher professional titles exercised more agency to implement advocated pedagogies and pioneer changes in class. This difference might be related to their variable identity commitments. Most experienced teachers with high professional titles tend to incorporate diverse identities as teachers, researchers, administrators, and policy practitioners who are most likely to pioneer educational changes at school or on indigenous levels.

**Table 2:** Teachers' agency differences among professional titles.

	**Mean**				**F**	**Sig**.
	**Primary** **(*n* = 120)**	**Intermediate** **(*n* = 150)**	**Associate** **senior** **(*n* = 64)**	**Senior** **(*n* = 4)**		
Teacher agency	3.99	4.09	4.10	4.72	4.410	0.005^**^
Intention	3.92	4.06	4.11	4.70	4.589	0.004^**^
Action	3.96	4.04	4.03	4.71	3.139	0.026^*^
Regulation	4.04	4.13	4.15	4.75	3.645	0.013^**^

The * and ** symbol indicates the values of p < 0.05 and p < 0.01 respectively.

Furthermore, a correlation analysis and a regression analysis were conducted to examine how well the scores of quantitative participants' agency were correlated with their reform-oriented beliefs. As shown in Table 3, there is a significant positive relationship between teachers' reform-oriented beliefs and agency. The findings indicate that teachers' beliefs significantly predict teachers' agentic performance, with teacher agency serving as the dependent variable and teachers' beliefs serving as the predictor, R^2^ = 0.412, *p* < 0.01.

**Table 3:** Correlations of the variables.

**Variables**	**Beta**	***t***	***p***	***R*** ^**2**^	***F***
Teacher agency	-	-	0.000^**^	0.412	244.949
Teachers' beliefs	0.642	15.651	0.000^**^		

** * p < 0.01*.

The interview data with the three case study participants further confirmed the close correlation among teachers' professional qualifications, reform-oriented beliefs, and agency presented in the survey data. That is, the qualitative data confirmed that the three teachers' past experiences and reform-oriented beliefs were closely linked to their agentic choices and actions, which was especially relevant to their sensitive perceptions of the current reform. In particular, the past experiences that the three teachers drew on when making a choice in accordance with policy mandates were often related to their perceptions, responsibilities, and participation in prior curriculum reforms. As the youngest teacher among them, Wang had obtained her Bachelor's degree as an English major at a prestigious foreign language university in China. She had not received teacher education prior to entering high school and had not experienced any previous national curriculum reforms. As shown in Extract 4, she has been struggling with the contradictions of students' passive learning, has clung to traditional teaching methods, and has been expected to adopt new approaches of pedagogy in the changing environment, which resulted in the incongruence between her reform-oriented beliefs and her actual behaviors in class. It may be inferred that superficial beliefs in curriculum reform and limited knowledge of educational theories are not sufficiently solid for teachers to make a professional shift beyond their immediate classroom experiential strategies.

#### Extract 4

Wang: Theoretical knowledge is certainly helpful for the quality of teaching, and the core competences were often heard when I attended academic lectures and peer colleagues' open classes. For me, however, I had little reference to the theories in my daily work and drew on only some advanced theories when participating in teaching contests. One reason is that the students' test scores in my class are the worst compared with other classes' students. The reform requirements are too difficult for them to meet.

Compared with Wang, Xu and Zhang are experienced teachers and the backbones of their schools. They were involved in previous curriculum reforms and acquired a wide repertoire of experience to cope with puzzles raised by educational changes, which might account for their adaptation to the current reform and confidence in their professional knowledge and pedagogic skills. During interviews, Xu and Zhang both stated that they have already published some reform-based research papers; meanwhile, they expressed strong desires to conduct more research on the basis of their pedagogical beliefs, practical knowledge, and self-reflection. The interviews revealed that deep insights into curriculum reform and critical reflection on creative pedagogies underpinned teachers' stronger sense of agency to engage in changes.

#### Extract 5

Xu: Since I had learned some new ideas, for example, the new term “cultural awareness” would replace the old term “cultural character” in the upcoming curriculum reform when communicating with some policy-makers and administrators of local educational office, I was prepared for the current reform. In the past, English teachers did pay enough attention to foster learners' Chinese cultural confidence, which had been modified in *xin kebiao*. Respect and appreciation for excellent cultures have been advocated to strengthen learners' patriotism and pride in Chinese historical culture.

#### Extract 6

Zhang: In the past 10 years, I have been committing to a series of training projects, namely, “Excellent Teachers', organized by the Municipal Education Department, where I kept learning and researching on advanced pedagogies; thus, my professional expertise has been greatly enhanced. This reform exerts pressure on me, but its policies are great. It centers on fostering students” integral competences rather than only judging them by academic performance; teachers are not simply imparters of knowledge but also helpers to scaffold students to establish correct values.

The results above suggest that Xu had focused on developing learners' cultural awareness and had reflected considerably on this term's connotation beforehand. Teachers are most likely to act as reform agents when their prior beliefs are in congruence with reform principles (Bonner et al., 2019). Xu acknowledged that it was impossible to reflect all the core competencies advocated by *xin kebiao* in assessment, while teachers should adhere to holistic educational goals in the long term and take into account students' academic excellence simultaneously, which inspired him to integrate valuable ideas of *xin kebiao* and high-stakes testing goals into his class. The interview data with Zhang confirmed that she exerted great efforts to foster learners' critical thinking by promoting academic knowledge and social justice to ensure that learners were equipped with a lifelong ability to learn and a sense of social responsibility. In general, the analysis of the three teachers' past experiences and beliefs revealed that teachers' experiences in career trajectories and reform-oriented beliefs are important prerequisites for alternative evaluation, decision making, and acting in a particular professional situation.

### Interaction With the Structural Environment

In this section, correlation analysis and regression analysis were conducted to examine how well the quantitative participants' perceived school culture correlated with teachers' agentic performance. A significant positive relationship was reported between perceived school culture and teacher agency, whose items of interpersonal condition were highly correlated with teachers' agentic performance. The findings shown in Table 4 indicate that perceived school culture significantly predicts teachers' agency level, R^2^ = 0.317, *p* < 0.01. These results might reveal that harmonious interactions with students and strong professional bonds with coworkers guarantee sustained agentic development for individual teachers.

Table 4Correlations and correlation of the variables.

**Table:** 

	**Perceived school culture**	**Interpersonal condition**	**School system**
Teacher	0.563^**^	0.528^**^	0.422^**^
agency

**Table:** 

**Variables**	**Beta**	***t***	***p***	***R*** ^2^	***F***
Teacher	-	-	0.000^**^	0.317	162.181
agency					
Perceived	0.642	12.735	0.000^**^		
school					
culture					

** * p < 0.01*.

Teachers' perceived school culture is another important factor in mediating teachers' agency during the reform. The qualitative analysis showed that the schools in which the three teachers worked had recontextualized policy mandates in different forms that had socially influenced their perceptions of their roles in the reform. Wang's data suggested that the school that she worked in concentrated more on teachers' examination accountability for evaluating students' learning and teachers' teaching, mainly based on test scores. Extract 7 also revealed that little intellectual support and interactions at the school level reduced Wang's possibilities of professional cooperation with colleagues. This evidence might partly explain Wang's great emphasis on students' concrete language skills in exam preparation.

#### Extract 7

Wang: Since English teachers at my school are overburdened with loads of classes, we have no stable time and space for regular teaching and researching sessions and simply gather together when there is a research activity held by the local educational office. Most of the time, we communicate *via* QQ group chat (a Chinese social medium) and share some teaching materials and resources that we believe to be useful. I truly hope that the school administrators could offer us more opportunities to learn advanced knowledge and skills in diverse ways.

Xu described that lately he had engaged in interschool English teachers' professional development training in association with curriculum reform. The teacher community mentioned in Extract 8 was organized to empower teachers to acquire a profound understanding of *xin kebiao*, which helped Xu to self-perceive as an active subject in educational changes. The data revealed that the creation of a learning environment for teachers who are enabled access to new educational theories and pedagogies in professional communities may enhance their sense of agency, confidence, and optimism regarding the reform.

#### Extract 8

Xu: My school has established a training program for teachers' professional development hand-in-hand with the Basic Education Institute of Jiangsu Academy of Educational Sciences, in which some chosen English teachers across the province are regularly organized to receive trainings for a week or two. During the session, I had chances to ask questions, discuss doubts and seek advice from experts and instructors. At the beginning, we all had no idea about the new curriculum reform, but we harvested many new theories when a series of trainings were completed.

Zhang mentioned in interviews that she and other faculty members in the English department met on the same workday each week to work on teaching plans and learn official documents together. Extract 9 demonstrates Zhang's enthusiasm for writing extended school-level textbooks and enriching teaching materials in a subgroup. This may indicate that intellectual exchanges occur through professional cooperation in different social activities, which create an opportunity for individual teachers to actively engage in the reform. It seems that Zhang not only perceived herself as an active performer of the reform but also performed in a larger picture of facilitating inexperienced teachers to take on new pedagogical innovations.

#### Extract 9

Zhang: School authorities require all English staff to attend regular weekly meetings to solve routine issues of daily work. We usually share some valuable resources, such as teaching plans, teaching design and PPTs, in giving and generous attitudes. To enrich students' cultural awareness, I set up a team in the department to build a school-level curriculum materials resource base, that aimed to work with other colleagues to intrigue learners' interests by containing more local cultures that they are familiar with in life.

### Aspirations for Professional Development

The projective dimension of teacher agency concerns both their long- and short-term work aspirations (Priestley et al., 2015). The interview data with the three teachers demonstrated that their future professional goals were closely linked to their agentic choices and actions during the reform. Wang said that it was absolutely necessary to “enact some advanced teaching theories and skills in class. However, for the students, entrance examination preparation to college upon which their future would be determined are most important, I don't dare to implement innovative pedagogies when *Gaokao* is approaching.” Additionally, she has undergone a professional dilemma and aspired to promote her professional learning by “participating in a Master program in the future and learning more knowledge of EFL educational theories.” It seems that her aspiration for a Master's degree regarding long-term professional goals articulated her hope of controlling her professional development trajectory with autonomy, which was in conflict with her short-term goal of pursuing higher scores and meeting students' academic standards set by school authorities. Comparatively, Xu's long-term goals in work were to “facilitate his students to gain more holistic knowledge and improve their language proficiency,” and his short-term goals were to “expand practical knowledge and conduct more reform-based research.” Zhang's long-term professional goals were to “explore deeply in educational theories and reinforce her students' social responsibilities, including how they could gain capability of life-long learning and how they could gain independence of thought and moral sense of judgment.” Her short-term professional goals were to “enhance professional expertise and academic competence through self-directed learning, cooperative learning and researching.”

The interview data and the teachers' published papers revealed that they had different expectations to conduct research. For example, Xu utilized mind maps in practical teaching and published academic papers based on his practical knowledge. Thus, he gave a future orientation for conducting more action research on relevant themes. Similarly, Zhang brought up her research interests as incentives to take on pedagogical changes. Extract 10 describes her research experience and agentic actions in focusing on activity teaching methods recommended in *xin kebiao*.

#### Extract 10

Zhang: Last summer, I attended a lecture given by an expert of curriculum reform on activity teaching methods, which raised my interests and inspired me to learn more about it. In the process of teaching practice guided by the method, I wrote an article and eventually published it, which gave me a great sense of achievement. I believe theoretical knowledge can instruct a teacher to supervise and evaluate his class, so I will continue to do research.

In all, Xu and Zhang were highly self-conscious that the class was inherently an “experimental field” where they enacted instrumental reform to thus promote their reflections on existing teaching practices. This evidence may indicate that teachers are most likely to act as reform agents when they can operate closely with positive interactions among their pedagogical beliefs, research interests, professional goals, and the reform policy.

## Discussion and Implications

In this study, the researcher endeavored to examine the agency of English teachers and analyze the influencing factors from an ecological perspective. Accordingly, the majority of teachers surveyed exhibited positive attitudes and beliefs about implementing the reform and noticeable inclinations toward change. Regarding the demographic characteristics, the findings confirmed that the scores of senior teachers' agency were much higher than those of other cohorts with lower ranks of professional titles. These results reveal that the imposition of national top-down educational policy from the macrolevel (i.e., the MOE) has strong impact on teachers' potential changes in their beliefs, behaviors, and competence in classroom practice. The teachers' abundant professional experience and resources may allow them to actively engage in policy implementation as motivational forces in its infancy.

Although the agency of English teachers demonstrated a relatively high implementation of policy mandates, their agency to act on changes is at variance with and is mediated by the personal qualities that they bring to their work. When the prescriptions and policy mandates are enacted at the microlevel (the classroom context), the qualitative study reveals that English teachers' high agency in the reform manifested in a series of sustained pedagogical change behaviors and reform-based reflections. Although the three case study teachers' ideas and beliefs are in congruence with those of the curriculum reform, they exhibited different agentic actions in the teaching process and pedagogical shift. Three forms of agency are found in this study: constrained agency, transformative agency, and progressive agency. More specifically, Wang demonstrated the constrained state of agency with notable inconsistency between positive reform-oriented beliefs and reversed actions in classroom practice. The problems in Wang's powerlessness in change and weak participation in the reform are predictable on the basis of her inexperienced practical knowledge and unsupportive school environment. Contrary to Wang, Xu and Zhang displayed considerable reflections on educational change and thus demonstrated themselves as active agents in consciousness and actions. The example of Xu supports the statement of being transformative by showing that his realistic intention of preparing the students for examinations and his ideal intention of promoting holistic education are integrated into his efforts to overcome the contradiction to then enhance teaching quality. In the same way, Zhang's progressive agency in the reform was manifested by actively constructing the expected identity imposed by educational changes and facilitating colleagues' growth in teaching.

**Figure 2:**
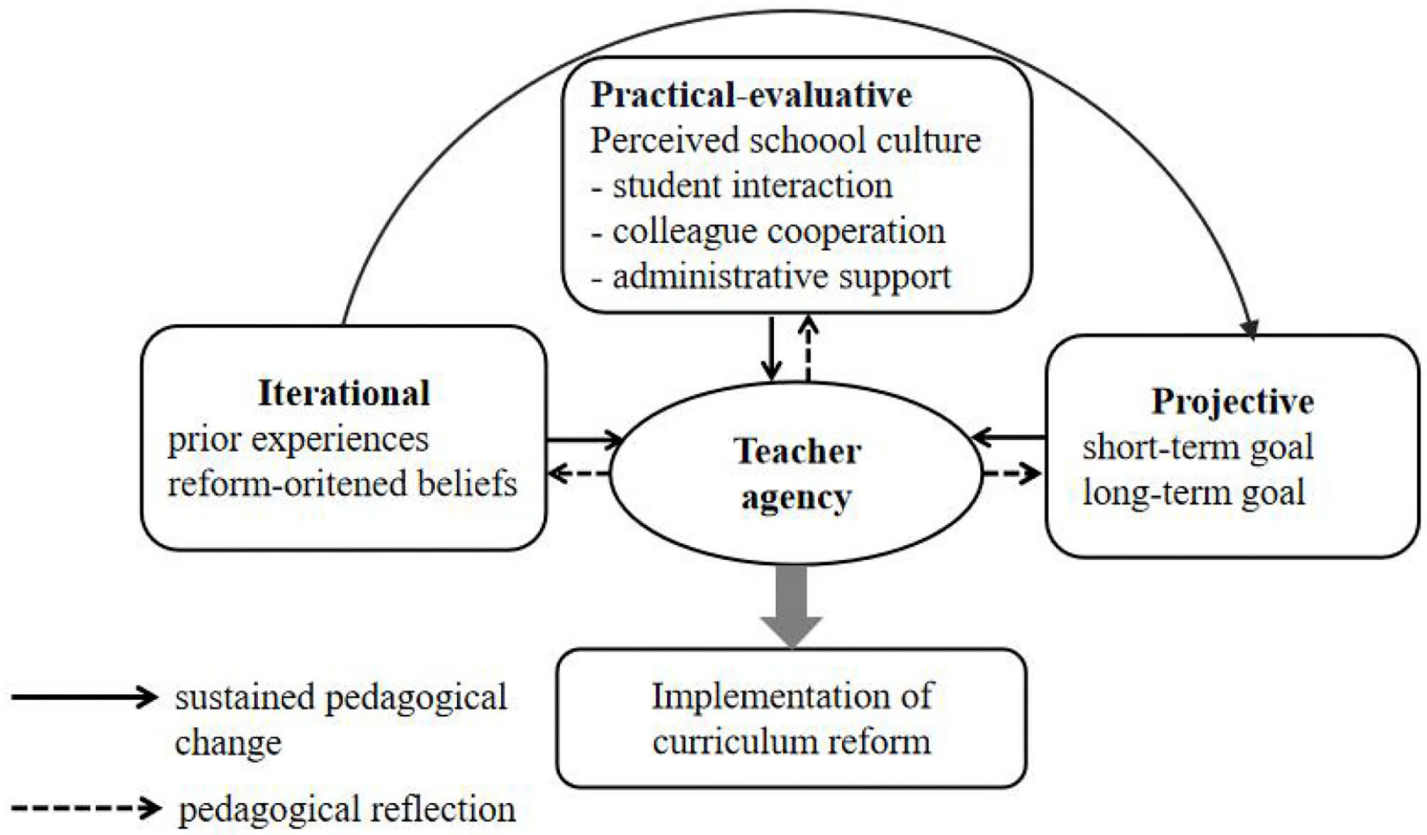
Formation of language teachers' agency in a curriculum reform context. Adapted from (2015), with permission from Bloomsbury Academic through PLS Clear.

These findings draw attention to how particular ecological conditions interconnect and interact with individuals, as captured in Figure 2.

Figure 2 presents a complex model of understanding the formation of language teachers' agency as a continuum that contains the three dimensions of iterational, practical-evaluative, and projective. Regarding the iterational dimension, as shown in Figure 2, prior experiences and reform-oriented beliefs were found to mediate teacher agency, which allows one to sustain previous practice or change on the microlevel. The implications of this model on the interplay between teacher agency and the iterational dimension that impacts it are as follows. First, this study supports the statement that particular experiences in a teacher's biographical dimension provide a strong ‘drive’ toward the future and make a clear difference in the here and now (Priestley et al., 2015). This study extends this perspective by finding that teachers' agency to change may originate from the recognition of one's agentic capacity that relies on transferring prior reform-related experiences into the present reform. A prior successful experience is likely to help teachers maintain confidence in their teaching ability (Chu et al., 2021; Liu et al., 2021). These prior successful experiences seem to enable teachers to have a clear awareness of implicit developmental opportunities, possible resources, and behavioral principles accompanied by educational changes and to thus take initiative to make reform-oriented choices and practical actions to transform in line with the reform. These findings also emphasize that the accumulated experiences and thoughts from the past are always intertwined with practical activities and are more significant than future professional goals in shaping teacher agency in curriculum reform in teachers' developmental trajectories. Second, reform-oriented beliefs are considered to be important factors to achieve agency in the iterational dimension. This is incongruous with the proposed pattern, which includes beliefs in the practical-evaluative dimension (Priestley et al., 2015) (see Figure 1), as the study found that teachers' reform-oriented beliefs that directed their actions were rooted largely in prior experiences and were more difficult to change than knowledge or practice during reform enactment. Although teacher beliefs always act in the present, belief change in teacher practice is difficult, slow, and often transient (Bonner et al., 2019). These findings also confirm that the agency to change is not activated by beliefs alone; a partial adoption of reform beliefs can also coexist with the agency of resistance toward the reform, but teaching and learning achievement gains for implementing reforms appear to be precursors of deep change in beliefs (Biesta et al., 2015; Naraian and Schlessinger, 2018; Bonner et al., 2019; Bao et al., 2020). For instance, the tension between Wang's stated beliefs and inconsistent behaviors in class was largely motivated by her perception of students' reactions and the safety of using innovative teaching methods in her classrooms. Since an insufficient experience with effective pedagogical strategies and practical knowledge is the primary cause of teachers' “willing spirit but weak power” phenomenon, it seems that policies prescribed in *xin kebiao* should be interpreted and implemented in more flexible and diverse senses rather than in narrow and rigid ways, which might lead to inappropriateness to the realities of the students. Thus, the critical question becomes how reform-related teacher educational programs could enable teachers to generate agency in challenging changes and gaining autonomy in contextual conditions. Third, this study implies that systematic reflections on prior experiences, future goals, and actions are seen as indispensable parts of teacher agency development. The three teachers interviewed reported their experiences of transferring familiar valuable acting principles into the reform context, in which reflective thinking was intertwined with their decision-making and action-taking in professional practices. Thus, reflection on action is considered to be the dominant activity for expanding practical knowledge and supporting teacher learning in general (Le et al., 2020), especially proactive reflection in evaluating aim accomplishment after experiences lay the groundwork for teacher learning in educational change.

For the projective dimension, future professional short-term goals and long-term goals on the microlevel were found to have been applied when evaluating alternatives, making decisions, and acting in a particular situation (Leijen et al., 2020). The results from the study concur with early research (Priestley et al., 2015), which suggested that teachers' short-term goals with respect to their day-to-day decision-making and teaching action are shaped by a perceived need to deliver enjoyable lessons and keep students engaged. For instance, examination preparation was prioritized when Wang exerted pedagogical practices in routine school work. Regarding long-term professional goals, the three involved teachers set purposes for improving teaching competence in different ways, which demonstrates that teachers are conscious of the imperative shifting of educational context in China and the need to transform their professional identity. Wang expressed aspirations to make progress in career development and be promoted as a senior teacher. Xu and Zhang mentioned digging deeper into the domain of the core competencies and developing wider social purposes of education. It is evident in these findings that when teachers' long-term professional goals are more congruent with ideas proposed by the policy, they will be more engaged in the reform. This suggests that educational innovation aligned with teachers' professional interests and expectations for the future can sustain and inspire their dedication to the whole process of curriculum reform. Therefore, in the early phase of curriculum reform, opportunities, social dialog, and time should be offered to teachers to become aware of their transformed roles and make sense of their views on the reforms (Vähäsantanen, 2015). It is also essential for policymakers and school administrators to create more opportunities for teachers to make a positive sense of professional development in the context of educational change.

Concerning the practical-evaluative dimension, a closer examination of how the variables of perceived school culture mediated the exercise of teacher agency on the meso level yielded additional evidence with implications for practice. The challenge of localizing the national curriculum should not be underestimated (Kennedy, 2013; Le et al., 2020). The results indicate that teachers' relationships with students, colleague cooperation, and administrative support strongly mediated teachers' practices during the whole reform period. Wang's previous intention to change was discouraged by students' inactive responses in class and insufficient support from the external environment. In contrast, Xu enhanced his sense of agency by participating in teacher communities organized by the school, and Zhang had access to more socially supportive interactions with the students and colleagues to develop relevant educational expertise. Therefore, the study confirms that whether school authorities attach importance to the reform by organizing training programs at the school level and interschool level and providing pedagogical resources for teachers affects their agency to a different degree. Given the deep-rooted tradition of examination-driven education in the Chinese context, localized school institutions always implement macrolevel curriculum reform as policy translators by changing the existing curriculum to various degrees based on their diverse financial conditions, deep-rooted culture, and hierarchical context. Notably, schools at the meso level interact with national educational policy, social ideologies, students' anticipations, and teachers' agency in a very complex way. Highly hierarchical school systems usually have close ties with less individual autonomy, which might be seen as a key competency of agency (Tao and Gao, 2021). Wang's constrained state of agency was largely accountable to her school context, where the regime of examination accountability prevailed and was dominant, while inadequate learning opportunities and professional help were offered. The finding echoes other published empirical results (Liu et al., 2016; Yang and Clarke, 2018; Poulton, 2020). Change requires both reculturing and restructuring of schooling (Biesta et al., 2015). However, the influence of schooling does not operate in a linear way on teachers because boosted agency may gradually drive the decomposition of traditional school culture and rebuild new culture, and vice versa. For example, Zhang constructed a teacher-learning community and created mutual support among colleagues through cooperation, which empowered her as a leading practitioner in the reform. Thus, suggestions for school institutions to relieve teachers' anxiety about educational change and enhance their agentic change include nurturing the role of reform leaders among teachers and providing them good opportunities for school-guided collaborative interaction, learning, and research. This study also calls for teacher education programs that concentrate on teachers' assessment literacy and help them find approaches in more profound ways to coordinate the inconsistencies between high-stakes examination preparation and holistic education.

## Conclusion

This study contributes to future research, as it demonstrates that English teachers' agency in response to the reform manifested in constrained, transformative, and progressive forms at the early phase of curriculum reform. In particular, the study found that teachers developed deep agentic changes through sustained pedagogical change and reflection on reform-based practices. Another important contribution of this study shows that the relationship between reform-oriented beliefs and teacher agency is more than linear. Teachers' agency to change is not activated or refrained by beliefs alone; other influential factors whose prior experiences, self-perceptions of transformative opportunities, accessible resources, and positive acting between pedagogical reflections and academic research also profoundly influence teachers' contextual agentic choices and actions.

To challenge the constraints on agency, regular learning opportunities need to be offered at the school-based level to foster teachers' curriculum reform literacy and assessment literacy, enhance their agency to take on positive changes, coordinate inconsistencies between high-stakes examination preparation and holistic education, and produce more autonomy in class practices. It is vital that a variety of indigenous school-based reforms be implemented to echo the national reform, which supports teachers in creating a space for critical reflection and negotiating more favorable identities in their professional trajectories. It is acknowledged that teachers' agency dynamically develops during different periods of curriculum reform (Priestley et al., 2015). In the future, longitudinal-designed research is needed to explore language teacher agency and provide more explanatory power for understanding agency in educational changes. Moreover, there is a lack of a sufficient amount of class observation that tracks interviewed teachers' agency development trajectories within classrooms. It is also worth examining teachers' discourse and actions in responding to the reform and enabling the student voice to be heard at the classroom level.

## Author's Note

LW is an associate professor in the School of Foreign Language, Guizhou University of Finance and Economics, Guizhou, China. LW interests include educational linguistics, language teacher development, and language education policy.

## Data Availability Statement

The original contributions presented in the study are included in the article/supplementary material, further inquiries can be directed to the corresponding author.

## Ethics Statement

Written informed consent was obtained from the individual(s) for the publication of any potentially identifiable images or data included in this article.

## Author Contributions

The author confirms being the sole contributor of this work and has approved it for publication.

## Funding

This work was supported by the Research Launch Project for Introducing Talents of Guizhou University of Finance and Economics (Grant No. 2020YJ029) and the Specialized Project of Key Discipline-Guizhou University of Finance and Economics (Grant No. 2020ZJXK08).

## Conflict of Interest

The author declares that the research was conducted in the absence of any commercial or financial relationships that could be construed as a potential conflict of interest.

## Publisher's Note

All claims expressed in this article are solely those of the authors and do not necessarily represent those of their affiliated organizations, or those of the publisher, the editors and the reviewers. Any product that may be evaluated in this article, or claim that may be made by its manufacturer, is not guaranteed or endorsed by the publisher.
